# Rapidly dissolving bilayer microneedles enabling minimally invasive and efficient protein delivery to the posterior segment of the eye

**DOI:** 10.1007/s13346-022-01190-x

**Published:** 2022-06-17

**Authors:** Yu Wu, Lalitkumar K. Vora, Ryan F. Donnelly, Thakur Raghu Raj Singh

**Affiliations:** School of Pharmacy, Medical Biology Centre, Queen’s University Belfast, 97 Lisburn Road, Belfast, BT9 7BL UK

**Keywords:** Dissolving bilayer microneedles, Transscleral delivery, Ovalbumin

## Abstract

The discovery of proteins that neutralise vascular endothelial growth factors, such as pegaptanib, ranibizumab and aflibercept, can inhibit the process of angiogenesis, thereby restoring eyesight in individuals with retinal vascular disorders. However, due to the posterior location and chronic nature of retinal diseases, a safe and effective intraocular protein delivery system is currently lacking. Thus, dissolving bilayer microneedles (MNs) with the potential to deliver proteins to the back of the eye in an efficient and minimally invasive manner were developed in this study. A model protein, ovalbumin (OVA), was incorporated into MNs fabricated from different polymers, including hyaluronic acid (HA), polyvinyl alcohol (PVA) and polyvinylpyrrolidone (PVP). Optimised PVA/PVP MNs were demonstrated to be robust enough to pierce porcine sclera with > 75% of the needle length penetrating the sclera and dissolving within 150 s. SDS-PAGE and OVA-specific ELISA revealed that the bioactivity of the model protein was maintained during the manufacture of MNs. In hen’s egg-chorioallantoic membrane test, MNs fabricated from all chosen polymers were classified as non-irritants. Furthermore, ex vivo permeation studies showed that optimised MNs could permeate 86.99 ± 7.37% of OVA through the sclera, twice that of the needle-free patch (42.16 ± 3.95%), highlighting the capability of MNs to circumvent physical barriers and promote protein delivery to the posterior segment of the eye. In this work, a novel, efficient and safe intraocular protein delivery system was successfully established.

**Graphical abstract:**

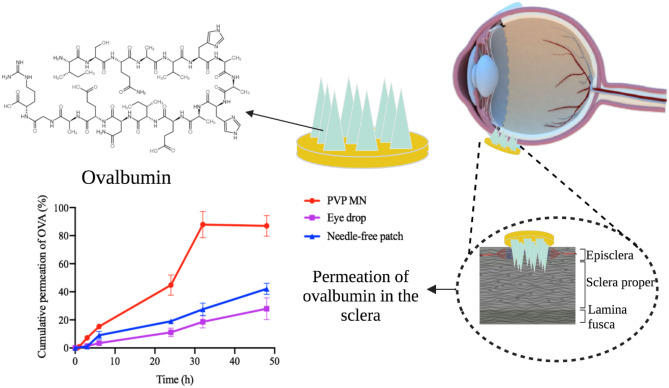

## Introduction

Retinal diseases such as macular degeneration, retinal detachment and diabetic retinopathy are leading causes of irreversible blindness in developed countries and significantly impact the individual patient’s quality of life [1]. Various biopharmaceutical drugs, such as aflibercept (Eylea®) [2], ranibizumab (Lucentis®) [3] and pegaptanib (Macugen®) [4], have been approved by FDA for retinal diseases treatment. Unfortunately, the investigation of effective and patient-acceptable protein delivery systems for alleviating posterior ocular diseases remains challenging. It is mainly due to the complex structure of the eye, especially the presence of ocular static and dynamic barriers, which form a fierce obstacle to protein delivery [5]. Additionally, the large molecular radius, charge, immunogenicity and enzymatic lability of protein drugs further complicate the intraocular delivery of proteins.

Currently, the mainstream route of protein administration to the back of the eye is intravitreal (IVT) injection. It has the advantages of bypassing ocular barriers and delivering macromolecules directly to the vitreous humour, thereby providing precise, immediate and effective treatment [6]. Although IVT injection can achieve higher ocular bioavailability of proteins than topical and systemic delivery methods, its high invasiveness cannot be ignored. Moreover, due to the chronic nature of retinal diseases, frequent dosing is required, which may lead to poor patient compliance and even the risk of severe complications such as cataracts, retinal detachment and elevated intraocular pressure [7]. Accordingly, there remains a necessity for novel drug delivery systems that can efficiently deliver macromolecules to the posterior segment of the eye in a more convenient and patient-friendly manner.

As a convenient, easily administered and minimally invasive device, microneedle (MN) has emerged as a reliable choice for transdermal and intraocular administration [8–10]. So far, various types of MNs have been developed and utilised for ocular drug delivery, namely solid, coated, hollow and dissolving MNs [11, 12]. However, as highlighted in previous MN-based investigations, silicon or metal MNs may be accompanied by limited drug permeability, poor reproducibility, brittleness and the possibility of accidental tissue damage (e.g. retinal damage and detachment)[13–15]. MN research has recently focused on dissolving MN prepared from FDA-approved biocompatible and biodegradable polymers, as this device has several distinct advantages[16, 17]. Firstly, the casting process for dissolving MNs is more straightforward and less expensive, enabling large-scale manufacturing [18–20]. Secondly, dissolving MNs can be applied by a one-step, self-administered process, which patients prefer over a two-step application process [21–23]. Additionally, dissolving MNs can bypass the biological tissue and form a depot in situ at the dosed site, thus enabling the localised and targeted delivery of therapeutic molecules and eliminating the generation of biohazardous sharps waste[24–26]. Despite these apparent benefits of dissolving MN and its extensive application in the transdermal administration of biomacromolecules [27, 28], studies on the use of dissolving MNs for the treatment of sight-threatening ocular disorders are still limited [29, 30]. This is possibly due to the challenges involved in the formulation and manufacturing of protein-loaded dissolving MNs for ocular drug delivery, such as protein waste, protein degradation and poor MN performance.

This work aims to develop and optimise dissolving MNs to provide an effective and patient-friendly option for the intraocular delivery of biomacromolecules. For this purpose, ovalbumin (OVA) was selected as a model protein for the typical anti-vascular endothelial growth factor (VEGF) drug ranibizumab because of their similar molecular weight (44 kDa compared with 48 kDa), hydrodynamic radius (5.8 nm compared with 5.5 nm), denaturation temperature (80 °C compared with 75 °C) and diffusion coefficient (7.76 × 10^–7^ cm^2^/s compared with 6.70 × 10^–7^ cm^2^/s) [31]. Furthermore, the use of OVA also enables the bioactivity analysis of protein by sodium dodecyl sulphate–polyacrylamide gel electrophoresis (SDS-PAGE) and enzyme-linked immunosorbent assay (ELISA). Accordingly, OVA was selected as the model protein for some degree of screening in early formulation development. A number of commonly used polymers for dissolving MN casting were investigated, including hyaluronic acid (HA, 200–400 kDa), polyvinyl alcohol (PVA, 31–50 kDa), polyvinylpyrrolidone K-29/32 (PVP, 58 kDa) and a mixture of PVA and PVP. Bilayer OVA-loaded MNs composed of these polymers were prepared and characterised for their mechanical strength, insertion depth and dissolution kinetics. Subsequently, the stability of the loaded protein was assessed by SDS-PAGE and ELISA to determine the appropriateness of the polymer utilised. Moreover, the ex vivo transscleral permeation profiles of MNs and conventional routes (i.e. eye drops and needle-free patches) were evaluated to compare their efficiency in facilitating macromolecule delivery.

## Materials and methods

### Materials

Albumin from chicken egg white lyophilised powder (referred as ovalbumin, OVA, lyophilised powder, ≥ 98%), bovine serum albumin (BSA), phosphate-buffered saline (PBS, pH = 7.4) tablets, sodium chloride (NaCl) and PVA (31–50 kDa) were purchased from Sigma-Aldrich (Dorset, UK). HA (sodium hyaluronate, 200–400 kDa) was obtained from Kewpie Corporation (Tokyo, Japan). PVP K-29/32 (58 kDa) and K-90 (360 kDa) were purchased from Ashland (Kidderminster, UK). Glycerol, AnalaR NORMAPUR, 99.5% was obtained from VWR International (Leicestershire, UK). All other chemicals were of analytical reagent grade.

### Fabrication of OVA-loaded dissolving bilayer MN

**Fig. 1:**
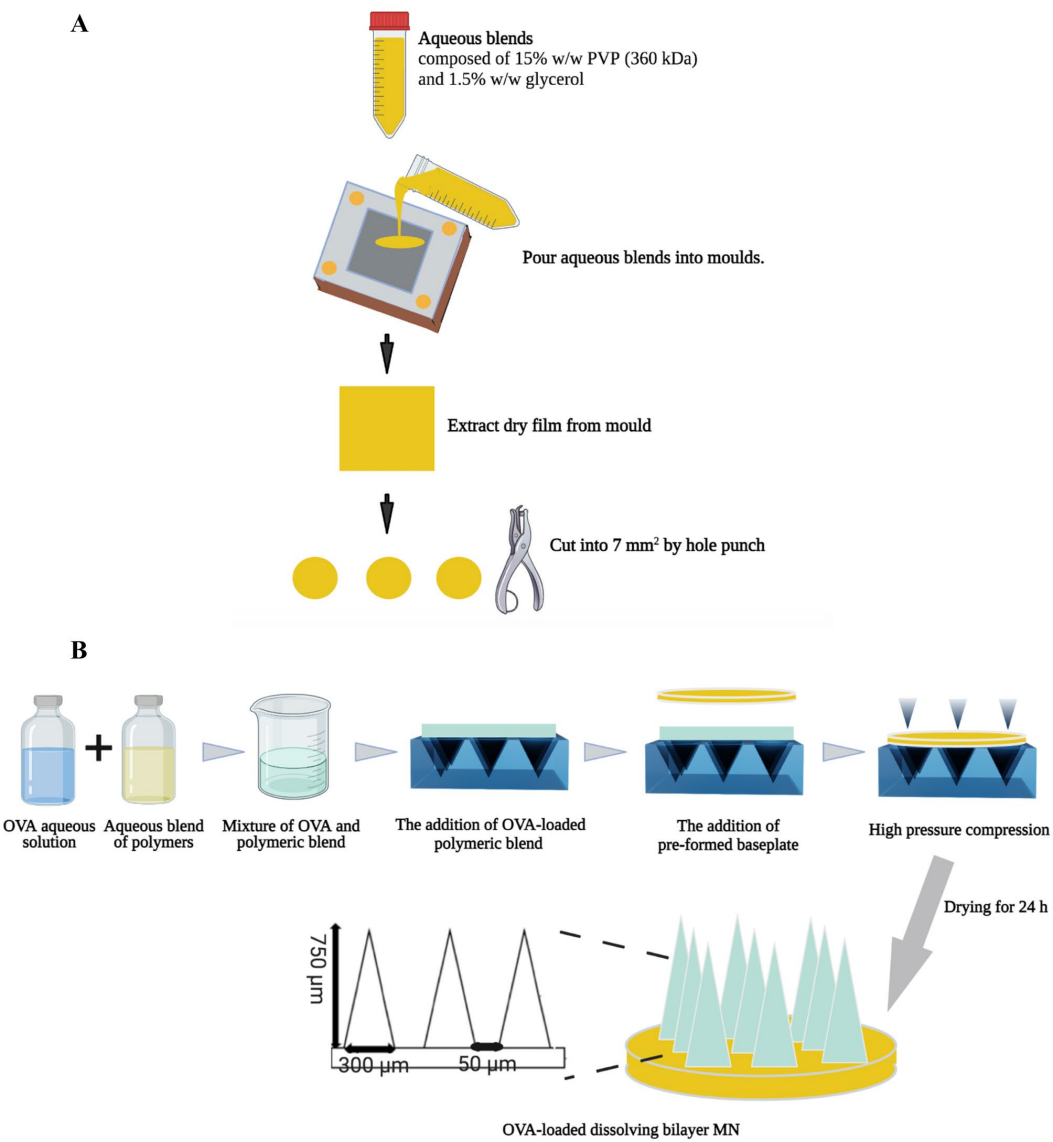
Schematic representation of the casting process of **A** pre-formed baseplate and **B** OVA-loaded bilayer MN

The fabrication of OVA-loaded dissolving MNs was divided into two main steps. Initially, aqueous blends of selected polymers were prepared and mixed with an appropriate amount of OVA. A 30% w/w stock of PVA 31–50 kDa was prepared by adding the required mass of PVA to water and then kept in an oven at 80 °C overnight until a clear gel formed. A 40% w/w aqueous solution of PVP K-29/32 was prepared by adding the required mass of PVP into deionised water and then stirring until a clear gel formed. A mixture of 15% w/w PVA and 20% w/w PVP was prepared by mixing the stock solutions of PVA and PVP in a ratio of 1:1. A 2.5% w/w stock solution of HA was fabricated by adding the required quality of HA to deionised water, followed by sonicating for 30 min to dissolve the HA. Subsequently, aqueous blends of selected polymers were mixed with the OVA aqueous solution. The compositions of the formulations prepared are listed in Table 1. After drying, the theoretical drug content of each MN based on the total weight of needle-part (~ 200 µg/array) was formulated to contain 100 µg OVA/array. Figure 1A indicates the manufacturing method of the baseplate layer composed of 15% w/w PVP K90 and 1.5% w/w glycerol. Before fabricating MNs, the baseplate layer was prepared and divided into small segments using a hole punch.

**Table 1:** Composition of the various formulations used to prepare MNs

Drug aqueous solution (% w/w)	Polymer (% w/w)
50% OVA	30% PVA 31–50 kDa
50% OVA	40% PVP K29-32
50% OVA	20% PVP K29-32 + 15% PVA 31–50 kDa
50% OVA	2.5% HA 200–400 kDa

Following the preparation of the pre-formed dry baseplate and drug-contained stock gels, 10 µL of the polymeric blend was accurately transferred onto a laser-engineered silicone conical mould (3 × 3 needles/array, 750 μm in height, 300 μm in base diameter and 50 μm interspacing) using a positive-displacement pipette (Positive-Displacement Pipette M100, Microman, Gilson, USA). The pre-formed dry baseplate was then placed on the back of the polymeric blend. Subsequently, the moulds were placed in a positive pressure chamber (Protima AT10 pressure tank, Richmond Scientific, Lancashire, UK) and a positive pressure of 5 bar was applied for 15 min to force the gel to fill the mould microprojections. The MNs were then dried at room temperature for 24 h and carefully removed from the mould. The manufacturing process of OVA-loaded MNs is shown in Fig. 1B. The prepared MNs were subsequently visualised under a Leica EZ4D digital light microscope (Leica Microsystems, Milton Keynes, UK) and a scanning electron microscope (SEM, TM3030, Hitachi, Krefeld, Germany) to measure the height and width of individual needles. Additionally, drug-free MNs were fabricated and recorded as blank MNs and compared with OVA-loaded MNs to investigate the effect of protein incorporation on MN characteristics.

### Mechanical properties of MNs

The mechanical test of prepared MNs with and without OVA was carried out using a TA-TX2 Texture Analyser (Stable Micro System, Surrey, UK), as described previously with slight modifications [32, 33]. Briefly, the MN was fixed to the movable Texture Analyser probe using double-sided paste tape with the needles pointing downwards. The probe was then pushed at 0.1 mm/s until the MN was in contact with a flat aluminium block, at which point it exerted a predetermined force of 3 N for 30 s. Individual MNs were observed and the average height of needles before and after the application of compression load was measured using the digital microscope. Hence, the reduction in MN height after compression could be calculated using Eq. (1), where H_BC_ is the height before compression and H_AC_ is the height after compression.

1 $$\%\mathrm{Reduction\;in\;MN\;height}=\frac{\mathrm{H}\text{BC }-\mathrm{H}{\text{AC}}}{\mathrm{H}\text{BC }} \times 100$$

### Evaluation of insertion ability of bilayer MNs

**Fig. 2:**
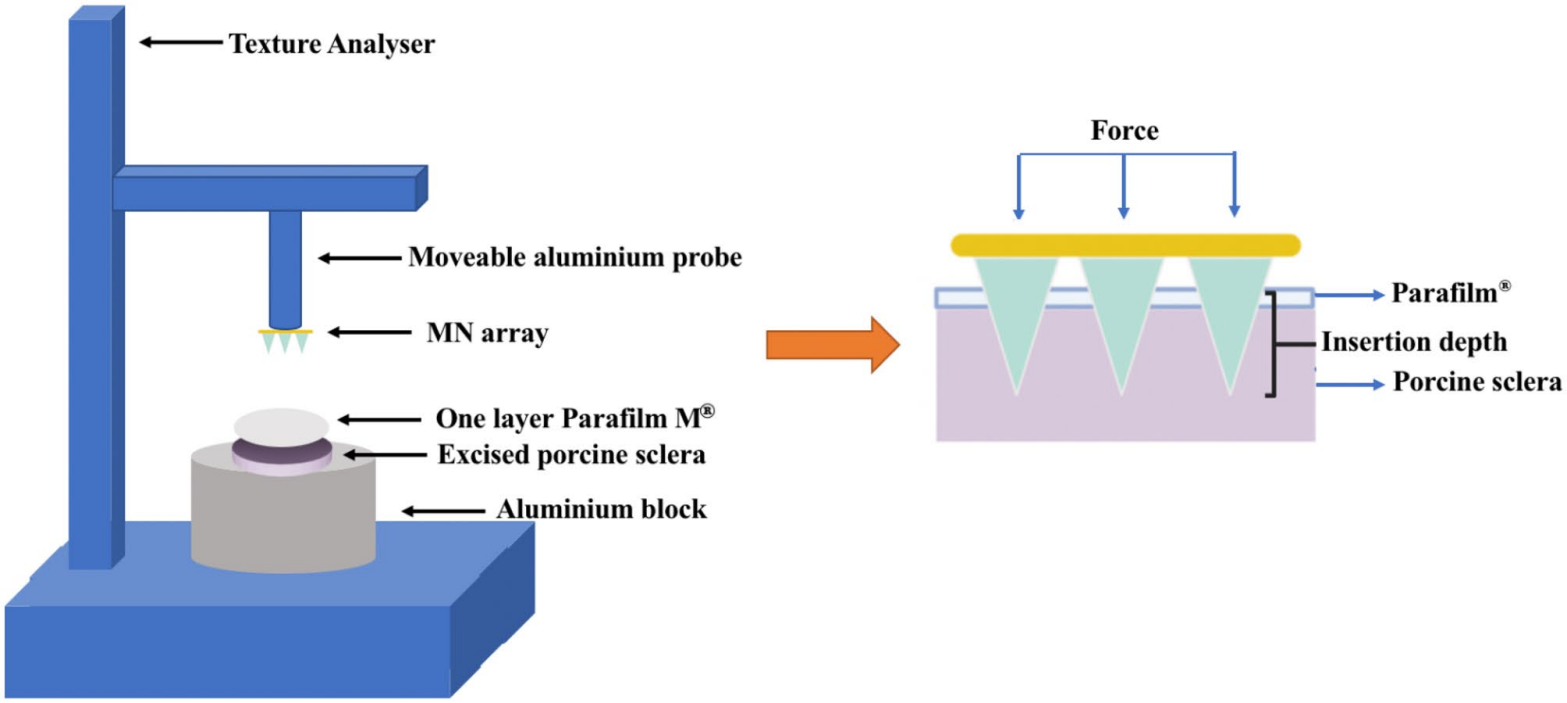
Schematic representation of the Texture Analyser set-up utilised to conduct the insertion test of MNs in excised porcine scleral tissue

The insertion study of the developed MNs was carried out using the Texture Analyser set-up shown in Fig. 2. Excised porcine scleral tissue was utilised as a model of human scleral tissue due to their similar general structure, thickness and water composition [20, 21]. Initially, the collected scleral tissue was hydrated and placed flat on a dental wax board. The scleral tissue was covered by a layer of Parafilm M® to prevent the rapid dissolution of the polymeric MNs, thereby enabling the accurate measurement of the insertion depth. After that, the MNs were compressed vertically into the parafilm-covered sclera using the Texture Analyser at a speed of 0.1 mm/s until the predetermined force of 3 N was achieved. After 30 s of compression, the probe moved upwards with a post-test speed of 1 mm/s. Optical coherence tomography (OCT, EX 1301 OCT microscope, Michelson Diagnostics, Kent, UK) was utilised to observe the insertion of the MN into the scleral tissue. The obtained image was then analysed using Image J® (National Institute of Health, Bethesda, USA) to measure the insertion depth. After insertion and observation, the MNs were carefully removed from the scleral tissue and visualised under the light microscope to evaluate the remaining height of the needle. The %reduction in needle height after insertion was calculated using Eq. (1).

### Determination of MN dissolution kinetics

The dissolution of blank and OVA-loaded bilayer MNs formulated from different polymers was investigated in situ in excised porcine scleral tissue, as described previously [34]. In brief, dissolving MNs were inserted into the collected scleral tissue and held for different time points (30, 60, 90, 120 and 150 s). After removal from the scleral tissue, the MNs were visualised under the digital microscope to measure the remaining heights of MNs. The %remaining MN height was calculated and recorded as MN height remaining vs time.

### Quantification of OVA content in MNs

In order to quantify the amount of OVA in the needle-part of each MN, the baseplate of the MN was held by a customised device and only the needle part was immersed in 2 mL of PBS (pH = 7.4) until all needles were dissolved. After complete dissolution, the sample was further diluted with PBS (pH = 7.4) and analysed by the ELISA method described in "Pharmaceutical analysis of OVA".

### Determination of OVA stability using SDS-PAGE

The stability of OVA released from the MNs formulated with different polymers was evaluated using SDS-PAGE. Initially, MNs were dissolved in PBS (pH = 7.4) and then 12 µL of NuPage® LDS sample buffer was added to 8 µL of dissolved MN solution. The samples were boiled at 70 °C for 10 min and then cooled to ambient temperature. Additionally, native OVA and fully reduced OVA were analysed and recorded as negative and positive controls, respectively. A NuPage® 4 to 12%, Bis–Tris, 1.5 mm, 10-well gel was rinsed three times using the running buffer (50 mL NuPage® and 950 mL deionised water) and placed in the XCell Surelock™ Mini-Cell gel running tank. Afterwards, 10 µL of each MN sample, control and diluted protein molecular weight marker (ladder) were loaded into each well of the gel. Samples were run for approximately 50 min at 200 V constant voltage and 220 mA maximum current, after which the gel was removed. Before putting in the gel fixer solution, the gel was rinsed with deionised water. After fixation, the gel was washed three times with a gel washer solution composed of methanol and deionised water. The gel was then incubated with cold silver nitrate solution (400 mg AgNO_3_, 152 µL formaldehyde and 200 mL deionised water) for 25 min. After that, the gel was rewashed with water to remove silver nitrate. The gel was then incubated with gel developer solution until the desired level of staining was acquired. Finally, the gel was transferred to gel stop solution and then the image was taken.

### Ex vivopermeation studies

**Fig. 3:**
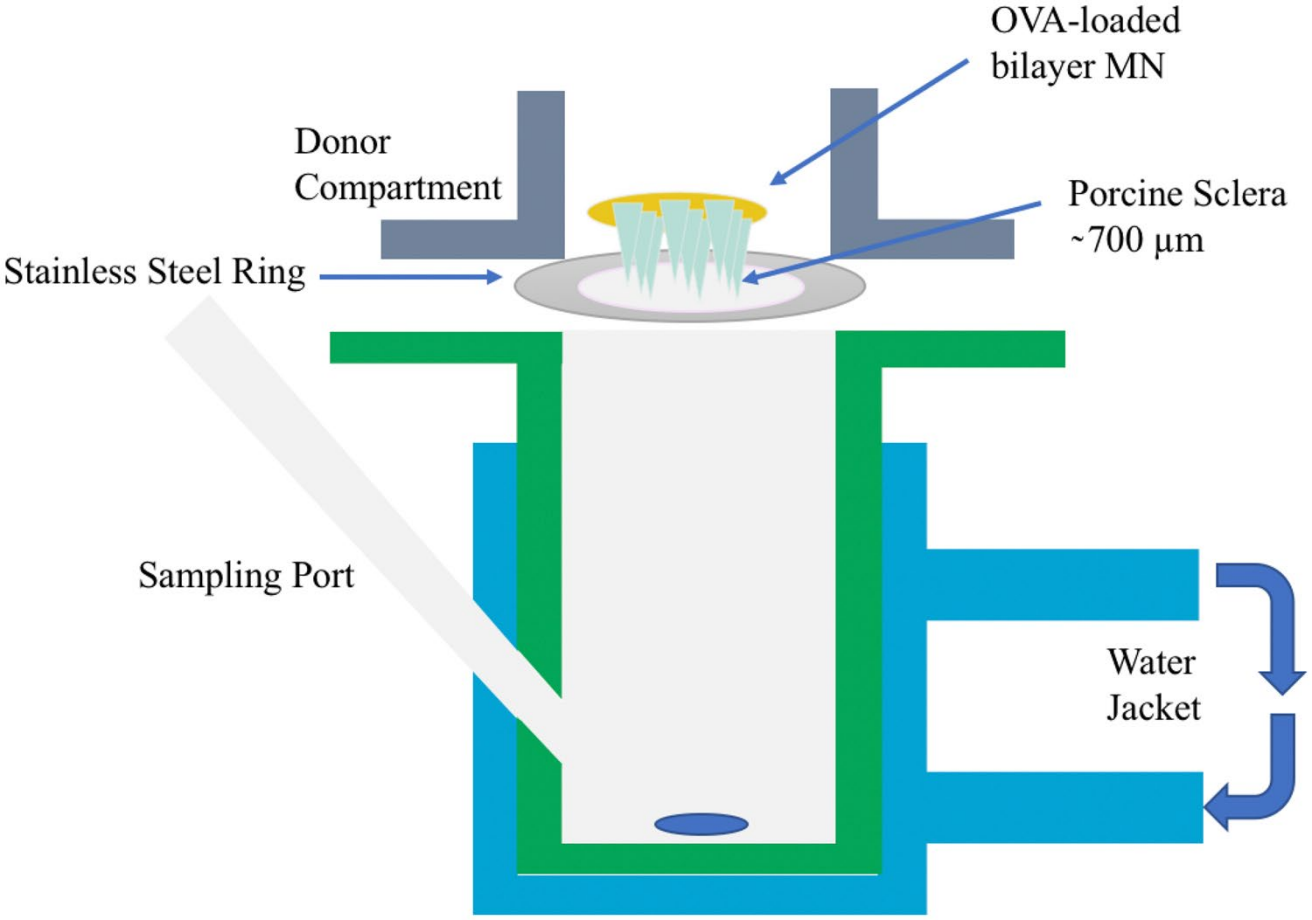
Schematic representation of the modified Franz-diffusion cell set-up for ex vivo permeation studies using excised porcine scleral tissue as a scleral model

Ex vivo permeation studies of MNs composed of different polymers were conducted utilising a modified Franz-diffusion cell set-up, as shown in Fig. 3. Before the permeation study, the scleral tissue was hydrated in PBS (pH = 7.4) for 2 h and then mounted on the donor chamber using cyanoacrylate glue. The receptor chamber was filled with 5 mL of pre-warmed PBS (pH = 7.4) and thermostated to 37 ± 1 °C. Subsequently, MNs were inserted into the sclera under finger pressure for 30 s. After 3 min of application, the MNs were removed from the scleral tissue while leaving the scleral tissue in the donor chamber for further incubation. Both the donor chamber and sampling arms were covered with Parafilm M® to prevent evaporation. Furthermore, 50 µL eye drops and needle-free patches with the same constituent as the optimal MN were prepared and compared with the dissolving MNs for their permeability profiles. Unlike MNs, which need to be removed after 3 min of application, the eye drops and needle-free patches were maintained in the donor chamber during the whole permeation investigation. At the respective sampling time points (1, 3, 6, 24, 32 and 48 h), 150 µL of the sample was retracted from the receptor chamber and replaced with the same volume of fresh PBS (pH = 7.4) using an elongated needle. The collected samples were stored in the fridge (4 °C) for further quantification using ELISA.

### Hen’s egg test-chorioallantoic membrane (HET-CAM) test

HET-CAM test was carried out according to the ICCVAM Recommended Test Method Protocol: HET-CAM Test Method to evaluate the eye irritation of OVA-loaded MNs formulated from different polymers [35]. The test samples were prepared by dissolving OVA-loaded MNs in 0.9% w/v NaCl. Afterwards, the solution of the MN was collected and filtered through a 0.2 µm syringe filter to sterilise the sample. Additionally, 0.9% w/v NaCl and 0.1 M NaOH were used as negative and positive controls, respectively. On day 0, the fertilised white Leghorn hen’s eggs were obtained from a local slaughterhouse. The eggs were placed in an advanced incubator (Brinsea Incubator Ltd., UK) with a rotating tray and incubated at 38.3 ± 0.2ºC and 58 ± 2% humidity until day 8. On the eighth day, the eggs were candled to check fertility and non-viable eggs were removed. In contrast, the eggs with the correct development of the embryo were returned to the incubator without rotation and maintaining the large end upwards. The eggs were candled again on the next day to mark the airspace. The eggshell above the air cell was carefully cut using a rotating dentist saw blade. Once the eggshell was removed, the inner membrane was moistened with 0.9% w/v NaCl. After removing the inner membrane, the highly vascularised chorioallantoic membrane (CAM) was revealed and treated with 0.3 mL aliquots of sterilised test samples and controls. Any emergence of irritation such as lysis, haemorrhage and coagulation over a 5-min period after the instillation of test samples would be documented.

### Pharmaceutical analysis of OVA

The OVA content in dissolving MNs and the cumulative amount of OVA in the receptor chamber of Franz cell set-up were quantified using the direct ELISA. The washing buffer was prepared by adding 0.05% v/v Tween 20 in PBS (pH = 7.4) and blocking buffer was prepared by dissolving 1% w/v BSA in washing buffer. After the preparation of reagents, 100 µL OVA samples and standards were coated into each well of a high-binding 96 well-plate and incubated overnight at 4 °C. The plate was then washed three times with 200 µL washing buffer to remove unbound OVA. Next, 200 µL of blocking buffer was added to each well to block the sites uncoated by OVA. After incubating for 20 min at room temperature, the plate was rewashed with washing buffer. The anti-OVA polyclonal antibody conjugated with biotin was diluted at 1:5,000 in blocking buffer and pipetted into each well in a volume of 100 µL. After 40 min incubation, washing was carried out three times as performed previously. The plate was incubated with the enzyme streptavidin–horseradish peroxidase diluted 1:10,000 in PBS (pH = 7.4) for 30 min and then washed with washing buffer. 100 µL of 3,3',5,5'-tetramethylbenzidine (TMB) was pipetted into the plate and incubated for 40 min under protection from light. Finally, a microplate reader (FLUOstar Omega, BMG Labtech, Weston Parkway, US) was used to evaluate the optical density at 650 nm.

### Statistical analysis

In this work, we compared two and more groups using Student’s *t*-test and one-way ANOVA by using GraphPad Prism® version 8 (GraphPad Software, San Diego, California, USA). In all circumstances, a difference was considered to be statistically significant when the *p*-value was less than 0.05 and probability value were recorded as * = *p* < 0.05, ** = *p* < 0.01 and *** = *p* < 0.001. Data are presented as means ± standard deviation (SD) of the mean from triplicate measurements.

## Results

### Microscopic observation of OVA-loaded dissolving MNs

In this investigation, OVA was used as a model protein to investigate the capability of MNs composed of different polymers to deliver the therapeutic macromolecules to the posterior segment of the eye. Selected polymers for casting dissolving MNs included PVA, PVP and HA, which have previously been demonstrated to be biodegradable, biocompatible and effective for ocular drug delivery [23, 24]. Table 2 summarises the digital microscope and SEM observations of MNs fabricated from different polymers. Each MN contained 3 × 3 individual needles. The height of each needle was found to be around 750 µm, the base width was approximately 300 µm and the interspacing was 50 µm, which was in close agreement with the scale of the mould. Furthermore, these images also indicate that MNs with sharp needles and intact structures could be successfully fabricated from the selected polymers.

**Table 2:**
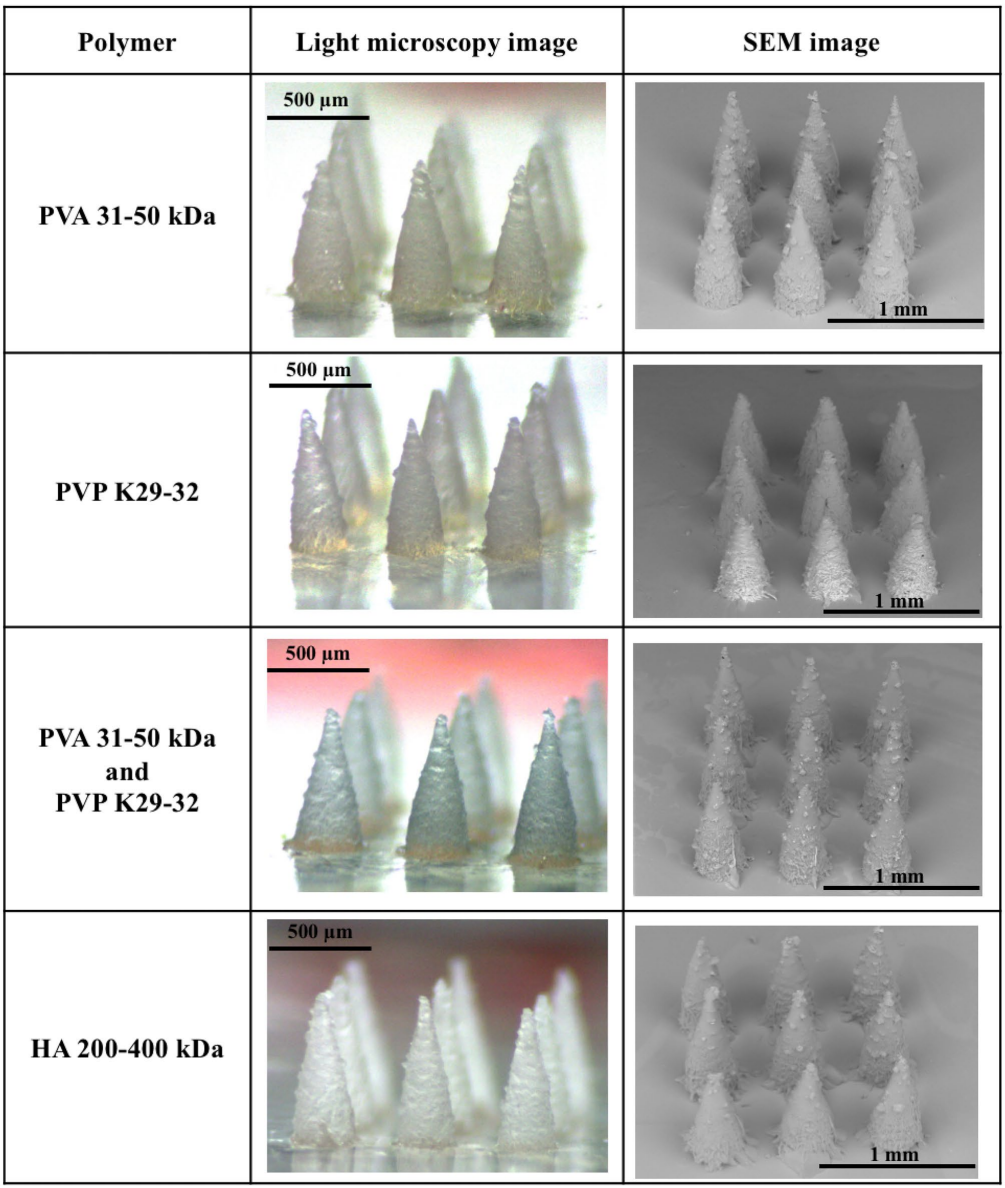
Summary of light microscope and SEM images of OVA-loaded MNs fabricated from various polymers.

### Mechanical characterisations of dissolving MNs

**Fig. 4:**
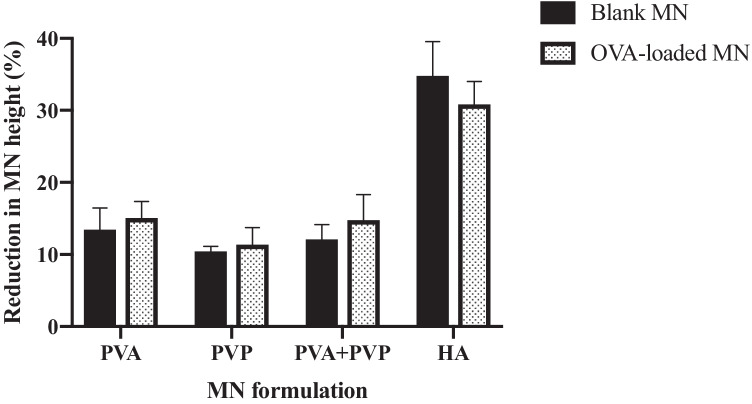
Comparison of percentage reduction in height between drug-free MNs (blank MNs) and OVA-loaded MNs prepared from different polymers after 3 N/array force compression (mean + SD, n = 3)

To ensure effective ocular drug administration, the developed MNs need to be sharp and strong enough to withstand the compression without breaking and be successfully inserted into the scleral tissue. Additionally, the incorporation of biomacromolecules may have either a weakening or a strengthening influence on the MN [36]. Therefore, it is crucial to assess the mechanical strength and insertion performance of the developed MNs. Figure 4 indicates the %height reduction of the MN after compression by a force of 3 N/array. Except for HA MN, which has the largest reduction in height (> 30%), MNs fabricated from other polymers exhibited sufficient mechanical strength with less than 15% height reduction observed following compression. In all cases, there was no significant (*p* > 0.05) difference between blank MNs and OVA-loaded MNs, suggesting that the incorporation of OVA was unlikely to adversely affect the MN robustness. Furthermore, after compression, the baseplate utilised to hold the MN needles was found to maintain its original shape and no cracks were observed, indicating that the pre-formed baseplate had sufficient mechanical strength to withstand the compression.

### Insertion capability of OVA-loaded dissolving MNs

**Fig. 5:**
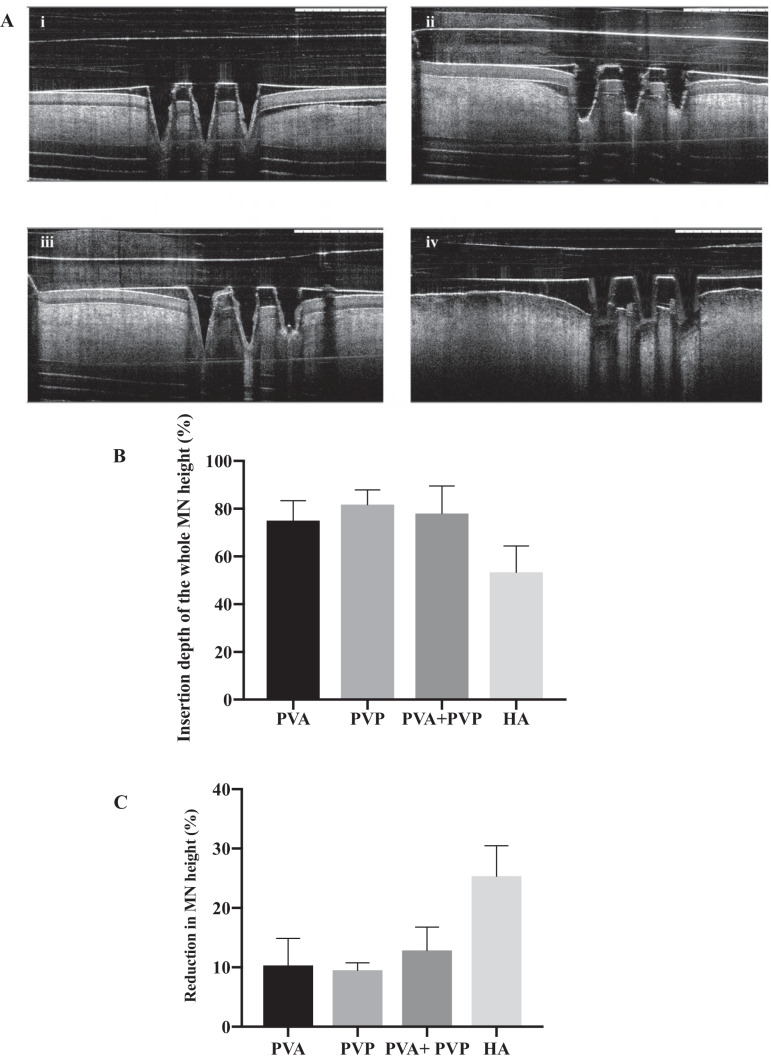
**A** The insertion results of OVA-loaded MNs prepared from different polymers, including (i) PVA, (ii) PVP, (iii) PVA + PVP and (iv) HA detected by OCT. **B** Percentage of whole MN needle length inserted into the excised porcine sclera. **C** Percentage of height reduction in OVA-loaded MNs after being inserted into the sclera under a force of 3 N/array (mean + SD, n = 3)

Different from the compression test, the insertion study was carried out using excised porcine scleral tissue. As shown in the OCT images of various MNs inserted into the sclera (Fig. 5A), there was a noticeable space between the scleral surface and the MN baseplate layer, suggesting that the needle length was not fully inserted into the sclera. However, this is not unexpected because it is challenging to insert the needle entirely into the sclera due to the viscoelasticity of biological tissue [25, 26]. Fortunately, with the exception of HA MNs, all other MNs exhibited good insertion capabilities with the insertion depth up to 75% of the MN height. After insertion and observation by OCT, the MNs were gently removed from the scleral tissue and observed under the light microscope to measure MN height reduction. Figure 5C shows that MNs fabricated from PVA, PVP and their mixture remained more than 85% in length after insertion. However, a significant height reduction (> 25%) was observed in HA MNs and only 53.32 ± 11% of the needle height was successfully inserted into the tissue. Given the fact that the insertion capability of the fabricated MN is highly correlated with its mechanical strength [27, 28], it is not surprising that the fragile HA MN exhibited poor insertion performance.

### Determination of MN dissolution kinetics

**Fig. 6:**
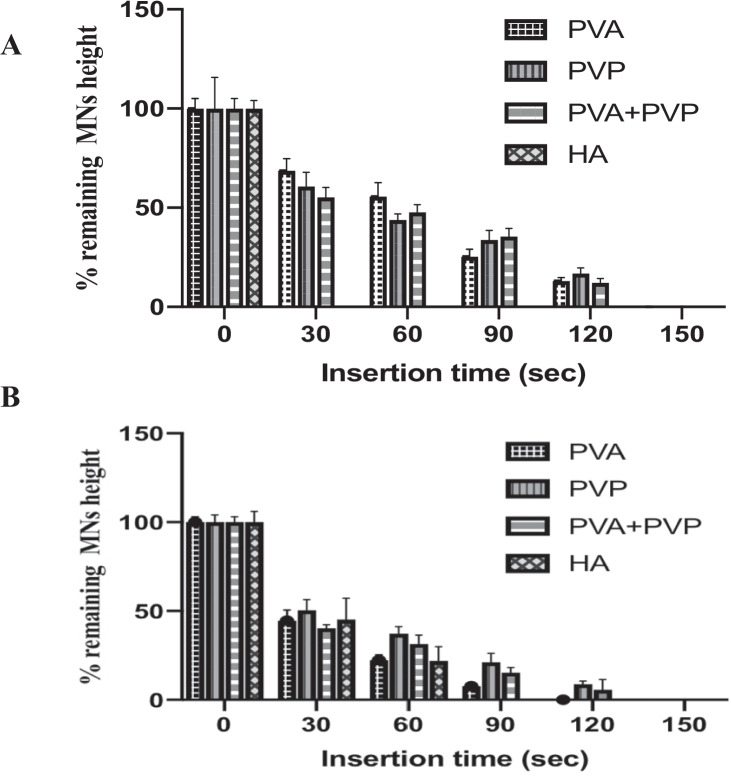
Percentage of MNs height remaining after the insertion of **A** blank MNs and **B** OVA-loaded MNs into the scleral tissue for predetermined time intervals (30, 60, 90, 120 and 150 s) (mean + SD, n = 3)

In order to determine the optimal time required to apply the fabricated dissolving MNs on the scleral tissue, dissolution studies of MNs composed of various polymers before and after OVA loading were carried out. Figure 6A, B summarises the time required for blank MNs and OVA-loaded MNs fabricated from selected polymers to dissolve in the scleral tissue. In relation to the blank MNs, except for HA MNs, which could be completely dissolved within 30 s, all other types of MNs took 2–3 min to dissolve. Given that the MN composed of HA has the lowest polymer concentration and HA has good solubility in water [37], this result is not surprising. After being loaded with OVA, MNs composed of PVA and PVP dissolved faster in the sclera, with more than half of the MN height dissolved within 30 s. In contrast, following the addition of OVA, the dissolution time of HA MNs was considerably prolonged from 30 to 90 s, as evidenced in Fig. 6B. Although the application time of HA MN was extended, which may result in decreased patient compliance, the MNs are still fit for the purpose. This is due to the fact that if MNs dissolve too quickly (< 1 min), the sharp tips of the MN may dissolve before they are fully inserted into the scleral tissue, which would result in the deposition of the drug payload on the scleral surface rather than within the sclera, leading to reduced bioavailability [38].

### Determination of OVA stability using SDS-PAGE

The purpose of this investigation was to develop a polymeric dissolving MN that can be used to load and deliver bioactive biomacromolecules to the back of the eye. Thus, it is essential to determine the stability of the OVA after being loaded into MNs. In this study, the MNs composed of various polymers were dissolved in PBS (pH = 7.4), after which the aqueous solution of the MN was withdrawn and analysed by SDS-PAGE. OVA not incorporated into the MN (free OVA) and fully reduced OVA were selected as the negative and positive controls, respectively, to show how the fragmentation pattern changes as OVA stability is compromised.

**Fig. 7:**
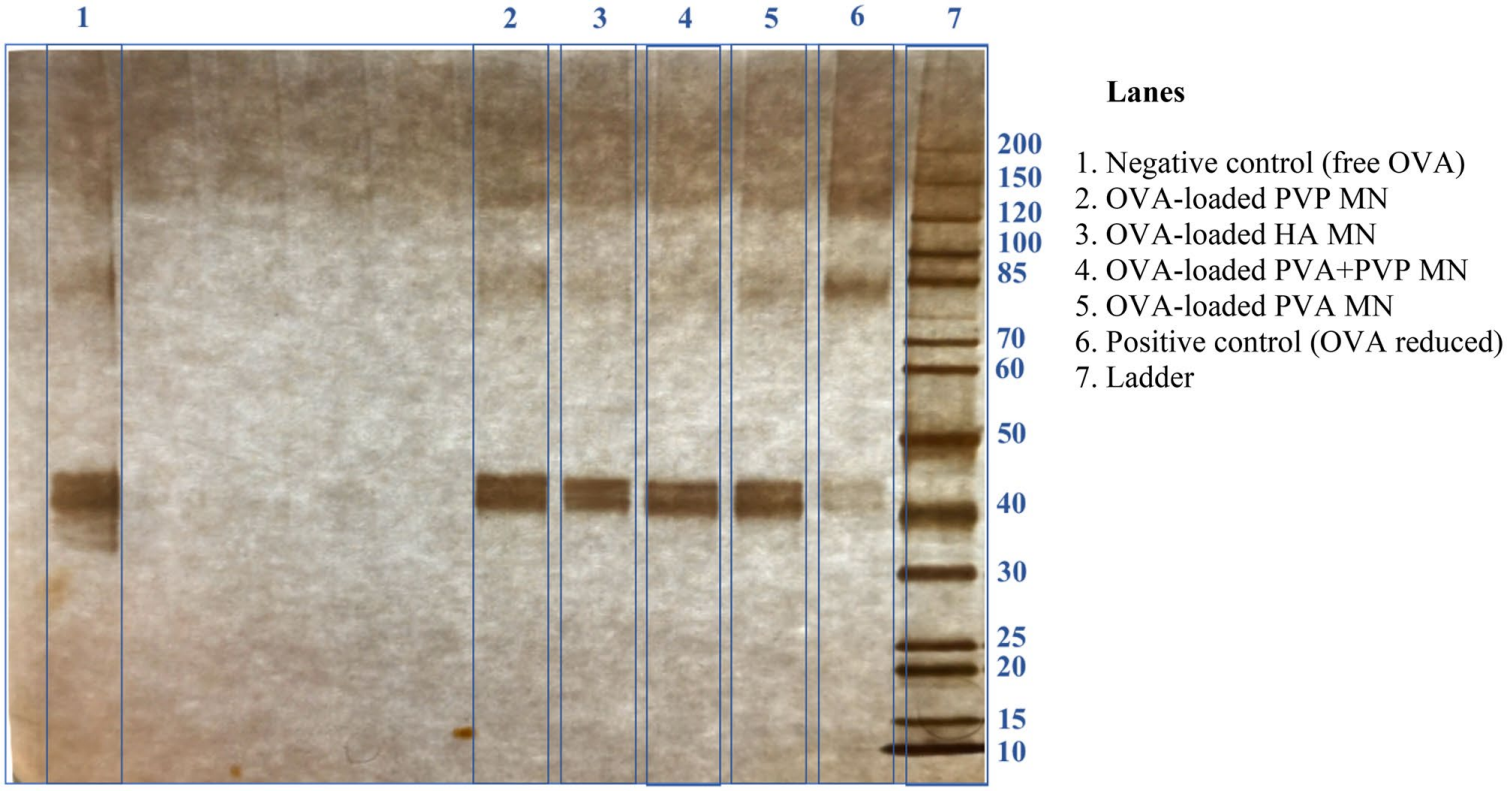
The image of SDS-PAGE analysis for free OVA, reduced OVA and OVA released from MNs made of selected polymers

Figure 7 displays the SDS-PAGE results of OVA released from MNs composed of different materials. A clear band at 44 kDa, just over the 40 kDa protein marker, was evident in both free OVA and OVA released from MNs. Conversely, this band disappeared in the lane for the positive control. Furthermore, in all lanes of MNs, except for the subunit around 85 kDa induced by OVA dimer, which is inherently contained in free OVA, no other bands could be observed, indicating that the incorporated OVA still maintained its molecular mass without aggregation or degradation.

### Determination of OVA content in MNs

Upon drying, the amounts of OVA in each PVA, PVP, PVA + PVP and HA MN array determined by ELISA were 105.66 ± 11.74, 110.96 ± 9.66, 94.52 ± 15.04 and 88.34 ± 13.12 µg/array, respectively. The difference between the amount of OVA recovered from each MN array and its initial addition (100 µg/array) was insignificant (*p* > 0.05), confirming the stability of OVA in these selected polymers. The results of ELISA were further underpinned by the findings of SDS-PAGE, whereby the manufacturing process of the bilayer MN and the incorporation of chosen polymers was shown to be harmless to the protein.

### Ex vivo permeation studies

**Fig. 8:**
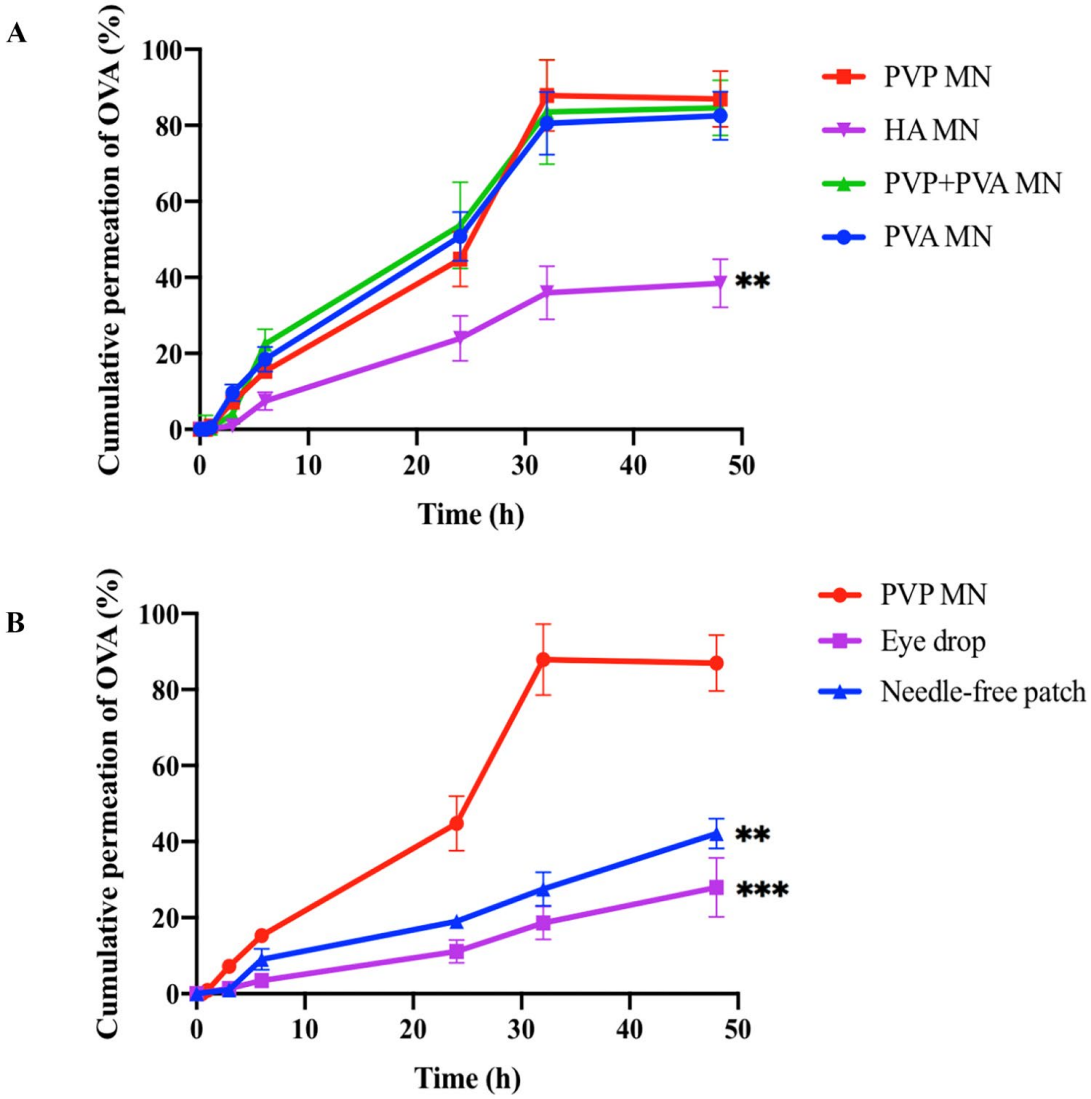
**A** Transscleral permeation profiles of OVA delivered by MNs fabricated from PVA, PVP, PVA + PVP and HA. The permeation profile of HA MNs is very significantly different (**) compared to MNs fabricated from PVP, PVA and the mixture of them. **B** ransscleral permeation profiles of OVA delivered by PVP MNs, eye drops and needle-free patches. The permeation profile of eye drop is very significantly different (**); needle-free patch is highly significantly different (***) compared to the PVP MNs (means ± SD)

The permeation study of OVA-loaded dissolving MNs was conducted using the Franz-diffusion cells set-up to ensure that the prepared MNs can penetrate the scleral tissue and deliver the drug to the posterior segment of the eye. Permeation profiles of MNs fabricated from selected compounds are shown in Fig. 8A. After 48 h, more than 80% of the drug load could be recovered from the receptor chamber upon delivery by the MNs containing PVA and PVP. This result confirms the capability of these MNs to successfully penetrate the scleral tissue and dissolve, thereby delivering most of the payload to the back of the eye. In contrast, HA MNs could only permeate 38.43 ± 6.38% of OVA, less than half of the other groups (*p* < 0.01). Leading on from these results, the selected HA was excluded from the candidate polymers for fabricating ocular dissolving MNs, whereas PVA, PVP and their mixture were recognised as better choices.

Subsequently, the PVP MN was selected as the model MN and compared with conventional routes of administration, including 50 μL eye drops and needle-free patches with the same composition as the OVA-loaded PVP MNs. The results are presented in Fig. 8B, which illustrates that in comparison with the aqueous solution of OVA, the introduction of the MN could significantly (*p* < 0.001) enhance the %cumulative permeation of OVA in the receptor chamber from less than 30% to 87%.

### Ocular irritation potential (HET-CAM test)

**Fig. 9:**
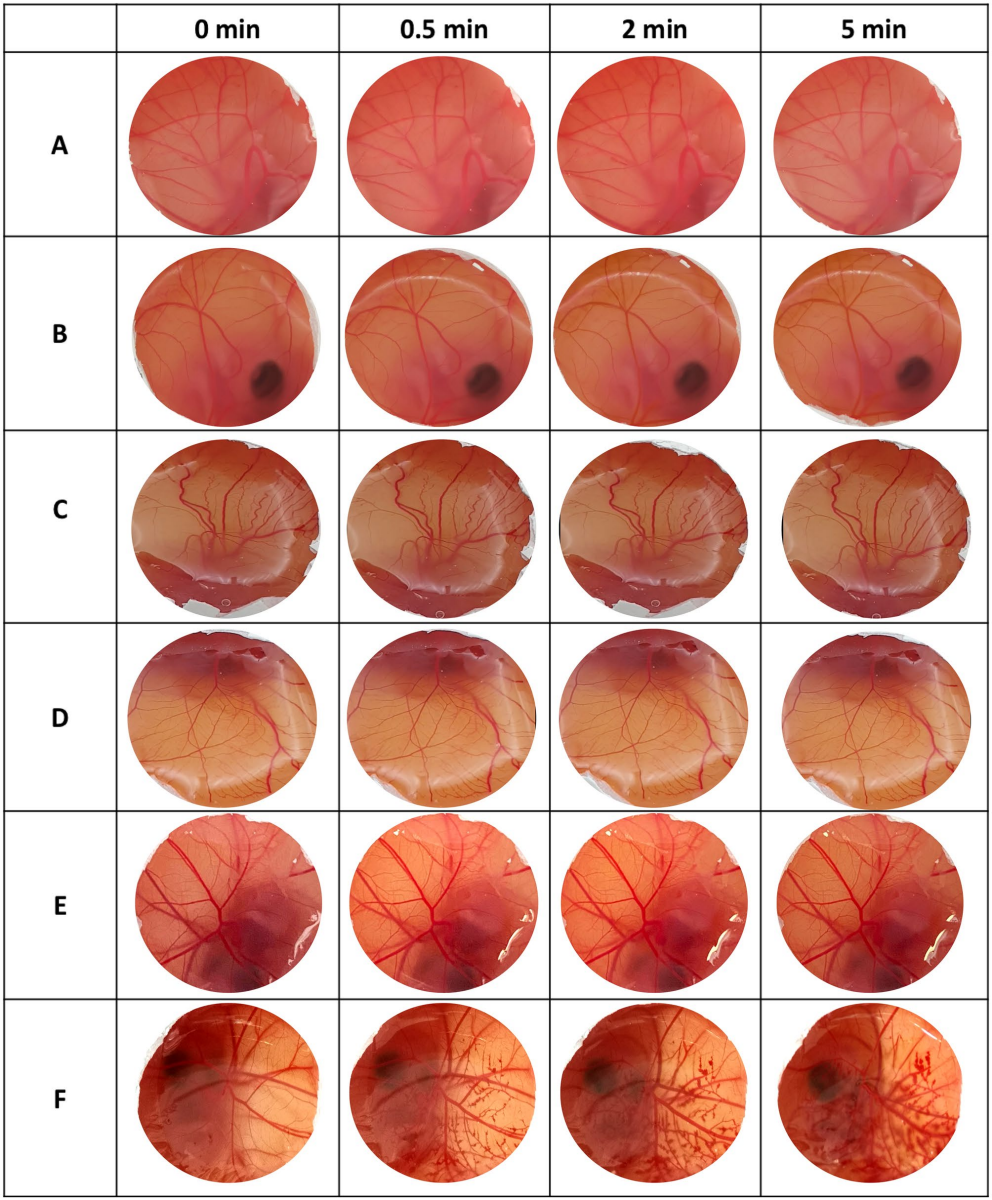
Evaluation of irritation potential of OVA-loaded dissolving MNs using the HET-CAM test. Observation of the vascular responses to assess the effect of **A** PVA MNs, **B** PVP MNs, **C** PVA + PVP MNs, **D** HA MNs, **E** 0.9% w/v NaCl (negative control) and **F** 0.1 M NaOH (positive control) on the CAM

Due to the delicate nature of the eyes, for drug delivery systems utilised to treat eye disorders, it is necessary to study the irritating potential of the formulation before testing in preclinical animal models. Accordingly, the eye irritation potential of the OVA-loaded dissolving MNs formulated from PVA, PVP, PVP + PVA and HA was determined using the HET-CAM test. The functional vasculature of the chicken placenta is used in this assay to simulate the ocular condition in vivo [39]. Versatility, ability to analyse numerous types of compounds (e.g. hydrophilic and hydrophobic molecules or solids), speed and ease of approach make the HET-CAM test a valuable alternative for assessing the eye irritation potential of developed MNs [40]. Following 5 min of exposure, the irritant response caused by samples and controls was recorded in images of the CAM (Fig. 9). As displayed, the instillation of 0.3 mL 0.9% w/v NaCl to the healthy CAM generated no visible response, ensuring that the assay conditions are safe for CAM. Conversely, the addition of NaOH induced a severe and immediate haemorrhage, which was classified as a severe irritant [35]. The obvious difference of CAM after treatment with the negative control and positive control confirmed the establishment of appropriate assay conditions. Within 5 min of observation, the eggs treated with OVA-loaded MNs showed no signs of vascular damage, revealing that all selected polymer formulations are non-irritating for in vivo use.

## Discussion

Due to the high invasiveness of conventional hypodermic needles and the requirement for frequent administration, IVT injection, which is the mainstay of treatment for retinal diseases, can potentially lead to devastating adverse effects. As a minimally invasive device, dissolving MNs can be an intelligent alternative to the conventional hypodermic needle and circumvent its aforementioned limitations. Anti-VEGF-based therapies are regarded as the first-line therapy to suppress neovascularisation and defend against retinal vascular disorders [41]. Commonly used anti-VEGF agents such as ranibizumab (Lucentis®), bevacizumab (Avastin®), aflibercept (Eylea®) and pegaptanib (Macugen®) are all biomacromolecules. Therefore, the development of dissolving MNs that can efficiently deliver protein drugs to the back of the eye is of paramount importance. However, the intraocular application of dissolving MNs to deliver biomacromolecules is still a novel topic and few investigations have been carried out on this. In light of this issue, the work outlined in this study selected OVA as the model drug for ranibizumab and developed OVA-loaded dissolving MNs for transscleral protein delivery [42, 43].

Previous work from our group has described the preparation of dissolving MNs loaded with fluorescein isothiocyanate–dextran with molecular weights of 70 kDa (FD70) and 150 kDa (FD150) by a one-step centrifugal casting approach [38]. The prepared MNs were proved to promote the delivery of macromolecules to the back of the eye. However, as demonstrated by McCrudden et al*.*, in the MN cast by the one-step centrifugal method, only 2% of the drug was localised in the needle part, whereas the majority of the drug (> 70%) was extracted from the baseplate layer [44]. This obviously has an impact on the drug delivery efficiency to the target tissue, as only the needle-part can be inserted into the tissue while the baseplate will be removed once the needle is dissolved. Accordingly, this manufacturing method always results in significant payload waste. Preliminarily, this casting method was applied to the model protein, which is cheap and easy to acquire, but for expensive anti-VEGF agents, it tends to be less cost-effective and such massive waste can even offset the dose-sparing effect of MNs. Therefore, the two-step casting and pre-formed dry baseplates were used to concentrate therapeutic molecules in the needle-part of MNs, thereby enhancing the bioavailability and delivery efficiency of the drug. Unlike other labour-intensive approaches that require complex manufacturing procedures to reduce protein waste and lead to poor reproducibility [45], two-step casting is a promising approach because it is simple, rapid and provides prospects for scaled-up production. 

In addition to the manufacturing approach, the choice of polymer is also critical in the formulation of the MN. The selected polymer must be sufficiently robust, rapidly dissolving, biodegradable and biocompatible. Therefore, in this investigation, several polymers were selected, including PVA, PVP and HA, which have been proven to be well tolerated in the eye and are widely used in ophthalmic drug delivery [23, 34, 35]. Due to its intramolecular rigidity, chemical stability, low manufacturing cost, excellent water solubility and low toxicity, PVP has been widely selected as the structural material for dissolving MNs. In the literature, MNs fabricated from PVP with different molecular weights (PVP K15, PVP K30 and PVP K29-32) were investigated [38]. Results demonstrated that PVP K29-32 could withstand higher forces with minimal height reduction and provide rapid dissolution in ocular tissues compared to PVP K15 and PVP K30. Thus, PVP K29-32 was selected as a candidate for the fabrication of MNs for ocular applications. Although MNs prepared from PVP K29-32 exhibited enough rigidity, the MN baseplates were found to be very brittle and fragile. Accordingly, more resilient and rigid baseplates were prepared using high molecular weight PVP (PVP K90) for all MNs tested in this study. Although PVP with very high molecular weights, such as PVP K87 (MW = 1000 kDa) are more difficult to excrete from the eye, the polymer in the baseplate would not be delivered into the tissue as only the needle part of MNs is inserted into the ocular tissue, so it is not considered harmful to the eyes. Furthermore, PVP K90 has been widely applied for the fabrication of the baseplate of dissolving MNs. In this regard, PVP K29-32 was used to prepare the needle part of MNs, while PVP K90 was chosen as the baseplate.

In order to determine the potential of the developed dissolving MNs to effectively deliver biomacromolecules to the posterior segment of the eye, a variety of parameters were evaluated, including the morphology, mechanical strength, scleral penetration, dissolution kinetics and permeation profile of MNs, as well as the stability of the incorporated protein. Furthermore, a key consideration in dissolving MNs developed for biomacromolecule administration is their ability to maintain the stability of incorporated proteins. In this investigation, the stability of OVA within different MNs was evaluated by SDS-PAGE and ELISA. As displayed in the results of SDS-PAGE, compared with free OVA, no discernible variations were observed in OVA released from the prepared MNs, suggesting that no degradation or aggregation occurred. The amount of OVA in each MN quantified by ELISA (> 88 µg/array) was in close agreement with the amount of OVA initially added (100 µg/array) (*p* > 0.05), indicating the casting procedure and selected matrix materials were compatible with the incorporated protein. Overall, these findings demonstrate the stability of OVA in chosen polymers and the viability of these MNs as vehicles for biomacromolecules administration.

The Franz-diffusion cell set-up was used to study the ex vivo permeation profile of OVA-loaded MNs. OVA-loaded MNs composed of various polymers were applied to excised porcine scleral tissue at a force of 3 N/array and their ability to deliver the drug across the sclera was evaluated by analysing the amount of OVA recovered from the receptor chamber. Herein, OVA-specific ELISA was used to quantify OVA, since it can not only distinguish OVA from interfering proteins adhering to porcine scleral tissue but also specifically count the amount of bioactive OVA. Compared with the other three formulations, MNs made of HA had the lowest permeation profile and could only deliver less than 40% OVA across the porcine sclera. This is unsurprising, as mechanical tests proved that HA MNs exhibited poor mechanical strength with more than 30% height reduction after compression. Furthermore, the insertion study highlighted the limited insertion capability of HA MNs. As evidenced by previous investigations, only drugs localised to the part of MN arrays inserted into biological tissue could be available for delivery [25, 46]. In this regard, the MN with the worst mechanical and insertion characteristics tends to exhibit poor delivery capability. Conversely, all other tested MNs were robust enough to pierce the scleral tissue, with more than 75% of total MN height (approximately 562 µm) successfully inserted, hence permeating a greater amount of OVA. The insertion depth of MNs was approximately 562 µm, while the thickness of the porcine scleral segment to which we applied the MN was around 735.6 ± 48.83 µm. The average thickness of human sclera was 670 ± 80 µm. This means that after the insertion of MNs and the biodegradation of the polymer matrix, the payload can be efficiently deposited into the bottom layers of porcine or human sclera, thereby bypassing the barrier function of scleral tissue and then diffusing to the adjacent choroid and retina [47]. Furthermore, since MNs could only penetrate to the bottom layer of the sclera and then soften and dissolve in the sclera rather than piercing the entire sclera, they avoid contact or damage to any highly sensitive tissues (e.g. choroid and retina), thus delivering the drug in a less invasive and painless manner. Accordingly, insertion depth in the sclera provided by the optimised OVA-loaded MNs can form a good compromise between delivery efficiency and patient compliance and is adaptable for efficient transscleral delivery.

In order to investigate the ocular delivery efficiency of MNs and conventional administrations, PVP MN was selected and compared with the permeation profiles of eye drops and needle-free patches composed of the same constituent. Following the application via the conventional routes, only less than 40% of OVA could be recovered from the receptor chamber, which was less than half of the amount of OVA permeated by PVP MNs. Given the dense connective nature of scleral tissue [38, 48], it is not surprising that it was difficult to use eye drops and needle-free patches to deliver macromolecules to the back of the eye. This finding was consistent with the data reported in our group’s previous work, in which the topical application of macromolecules (FD70 and FD150) with the aim of permeating through the scleral tissue was found to be challenging. In contrast, the introduction of dissolving MNs could dramatically improve the transscleral delivery efficiency of these macromolecules [38]. Furthermore, in practice, the retention time of eye drops and needle-free patches is limited and is unlikely to last 48 h [49]. Hence, the actual permeation profiles of these conventional routes will be drastically reduced and much lower than the dissolving MNs. Taken together, the results presented here, combined with evidence reported in other published studies, suggest the vital role of dissolving MNs in bypassing the ocular tissue barriers and promoting the transscleral permeation of macromolecules.

The protein-loaded bilayer MNs designed in this investigation enable the self-administration of biological macromolecules to treat retinal diseases. Unlike conventional hypodermic needles that require trained medical staff and often cause discomforting eye reactions, this novel device is easy to handle and user-friendly. Protein waste can be eliminated as the therapeutic molecules are specifically concentrated in the needle-part. Moreover, the developed OVA-loaded MNs were demonstrated to be safe for ocular administration and could significantly promote the permeation of biomacromolecules through the scleral tissue. Accordingly, the designed device provides a convenient and less invasive choice for the intraocular administration of biomacromolecules.

## Conclusion

In this investigation, the protein-loaded dissolving bilayer MN was successfully developed to facilitate the transscleral delivery of biomacromolecules. The pre-formed baseplate and positive pressure were used to concentrate the protein in the needle-part to reduce loss of protein. The protein released from MNs manufactured via this approach was demonstrated to be bioactive by conducting SDS-PAGE and ELISA. Furthermore, several biodegradable and biocompatible polymers were screened for MN fabrication and characterised for mechanical strength, insertion depth, dissolution kinetics and permeation profiles. Based on these evaluations, PVP, PVA and the mixture of PVA and PVP were revealed to be suitable for casting MNs with excellent physical properties and sufficient intraocular delivery efficiency. The ex vivo permeation studies demonstrated that the introduction of MNs enabled rapid and efficient delivery of the protein to the posterior segment of the eye. In conclusion, the developed protein-loaded bilayer MNs provide a novel and promising option for treating retinal disorders in an effective and minimally invasive manner.

## Data Availability

All the data and materials used are freely available.

## Declarations

### Research involving human and animal participants

This study was performed without use of animals and human volunteers. Therefore, this is not applicable.
